# Supplementary material for: Structure of HIV-1 gp41 with its membrane anchors targeted by neutralizing antibodies
Source: eLife. 2021 Apr 19;10:e65005. doi: 10.7554/eLife.65005 (PMC8084527; doi:10.7554/eLife.65005)
Supplement: Table 1—source data 1. [file elife-65005-table1-data1.docx]

**Table 1- source data 1**. Env pseudoviruses

| **HIV-1 Envelope** | **HIV subtype** | **Neutralization Tier** | **Accession Number** |
| --- | --- | --- | --- |
| NL4_3 | B | 1 | U26942 |
| MN-3 | B | 1 | AY669737 |
| BaL.26 | B | 1 | DQ318211 |
| SF162.LS | B | 1a | EU123924 |
| SF162P3_cl2-4 | B | 2 | AY988107 |
| JR-FL | B | 2 | AY669728 |
| JR-CSF | B | 2 | AY669726 |
| QH0692.42 | B | 2 | AY835439 |
| THRO4156.18 | B | 2 | AY835448 |
| SC422661.8 | B | 2 | AY835441 |
